# The expression of *AURKA* is androgen regulated in castration-resistant prostate cancer

**DOI:** 10.1038/s41598-017-18210-3

**Published:** 2017-12-21

**Authors:** Kati Kivinummi, Alfonso Urbanucci, Katri Leinonen, Teuvo L. J. Tammela, Matti Annala, William B. Isaacs, G. Steven Bova, Matti Nykter, Tapio Visakorpi

**Affiliations:** 1 BioMediTech Institute, Faculty of Medicine and Life Sciences, University of Tampere, Fimlab Laboratories, Tampere University Hospital, Tampere, Finland; 2 Department of Urology, Tampere University Hospital and Faculty of Medicine and Life Sciences, University of Tampere, Tampere, Finland; 3 The James Buchanan Brady Urological Institute, Johns Hopkins University School of Medicine, Baltimore, Maryland 21287 USA; 4 Centre for Molecular Medicine Norway, Nordic European Molecular Biology Laboratory Partnership, Forksningsparken, University of Oslo, Oslo, Norway; 5 Department of Molecular Oncology, Institute for Cancer Research, Oslo University Hospital, Oslo, Norway; 6 Department of Tumor Biology, Institute for Cancer Research, Oslo University Hospital, Oslo, Norway; 7 K.G. Jebsen Inflammation Research Centre, University of Oslo, Oslo, Norway

## Abstract

Although second generation endocrine therapies have significantly improved survival, castration-resistant prostate cancer (CRPC) cells are eventually able to escape available hormonal treatments due to reactivation of androgen receptor (AR) signaling. Identification of novel, non-classical and druggable AR-target genes may provide new approaches to treat CRPC. Our previous analyses suggested that *Aurora kinase A* (*AURKA*) is regulated by androgens in prostate cancer cells that express high levels of AR. Here, we provide further evidence that AURKA is significantly overexpressed in AR-positive CRPC samples carrying amplification of *AR* gene and/or expressing AR in high levels. We also demonstrate androgen-induced AR binding in the intronic region of *AURKA*. The expression of *AURKA* is increased upon androgen stimulation in LNCaP-ARhi cells that express high levels of AR. The growth of the cells was also significantly inhibited by an AURKA specific inhibitor, alisertib (MLN8237). Together, these findings suggest that the expression of *AURKA* is regulated by androgen in prostate cancer cells that highly express AR, emphasizing its potential as a therapeutic target in patients with CRPC.

## Introduction

Androgens and androgen receptor (AR) are known to be important drivers of prostate cancer progression^1,2^. AR is a ligand-dependent transcription factor that is a member of the steroid hormone receptor family, and it binds to androgen responsive elements in DNA to regulate androgen responsive genes^3^. The current standard treatment of advanced prostate cancer is androgen-deprivation therapy (ADT)^1,3^. Eventually, most ADT-treated patients develop castration-resistant prostate cancer (CRPC), which expresses functional AR. Most CRPC patients respond to second-line hormonal therapy, such as enzalutamide or abiraterone, for a limited period^1,3–5^.

Almost all prostate cancers are AR-positive^2^. The incidence of de novo AR-negative small cell carcinomas with signs of neuroendocrine differentiation has been reported to be between 0.5 to 2% of all prostate cancers^6,7^. In addition, some AR-positive prostate cancers transdifferentiate during ADT to an AR-negative, neuroendocrine type of prostate cancer (NEPC). It has been suggested that 10 to 25% of advanced AR-positive prostate cancers will become AR-negative NEPC^2,7^. We have recently demonstrated that the frequency of AR-negative cancers is 1.5% in locally recurrent CRPC and 7% in CRPC metastases^8^. AR-positive prostate cancers and AR-negative NEPCs seem to have, at least in some cases, the same clonal origin because they share the same molecular alterations, such as ERG rearrangements^6,7,9,10^.

Aurora kinase A (AURKA) is a serine-threonine kinase that functions in mitotic spindle formation and chromosome segregation^11–16^. It has oncogenic properties when aberrantly expressed, inducing aneuploidy and cell transformation^11,13,15,16^. During mitosis, AURKA localizes to the centrosomes and mitotic spindle poles, and it associates with other co-activators that determine its exact function^14,16^. AURKA has been shown to be highly expressed, especially in AR-negative NEPC^6^ and in basal cell-like breast cancers^17^.

We have previously found that *AURKA* is upregulated in prostate cancer cells that overexpress AR (VCaP and LNCaP cells stable-transfected with AR) under DHT stimulation and is overexpressed in CRPC^18,19^. Additionally, chromatin immunoprecipitation sequencing (ChIP-seq) studies by us and others have indicated that LNCaP and VCaP cells have a putative androgen receptor binding site (ARBS) in the promoter and intronic region of *AURKA* ^20–22^. A recent study by Pomerantz *et al*.^23^ suggested that the intronic ARBS of *AURKA* is a prostate cancer-specific AR binding event. Here, we validated *AURKA* as a novel, non-classical androgen responsive gene in CRPC cells highly overexpressing AR. We also studied the expression of AURKA in clinical prostate cancer samples and its association with the AR expression levels. Finally, we studied the effect of AURKA specific inhibition in CRPC cells highly expressing AR.

## Results

To find novel, prostate cancer specific clinically relevant AR-target genes, we integrated earlier published data based on gene expression^18^ in clinical prostate cancer specimens with AR-ChIP-seq data^18–22,24^ (Supplementary Figure S1). In our earlier work^18^,we identified a set of 54 genes which we prioritized based on association of their expression with disease outcome and presence of prostate cancer specific AR binding sites (ARBSs) (Supplementary Table S1). Gene-wise Kaplan-Meier re-analysis of the Taylor *et al*.^24^ dataset showed association of positive expression with outcome for ten genes with *AURKA* showing best association (Supplementary Table S1). *AURKA* was found to be overexpressed in prostate cancer, especially in CRPC specimens (Supplementary Figure S2). We have also previously shown that the expression of *AURKA* is increased with androgen stimulation in LNCaP-ARhi cells expressing high levels of AR^18^ (Supplementary Figure S2), and ChIP-seq analyses have indicated a putative prostate cancer specific ARBS in the promoter as well as in the intronic region of the gene^20–23^ (Supplementary Table S1, Supplementary Figure S3).

**Figure 1:**
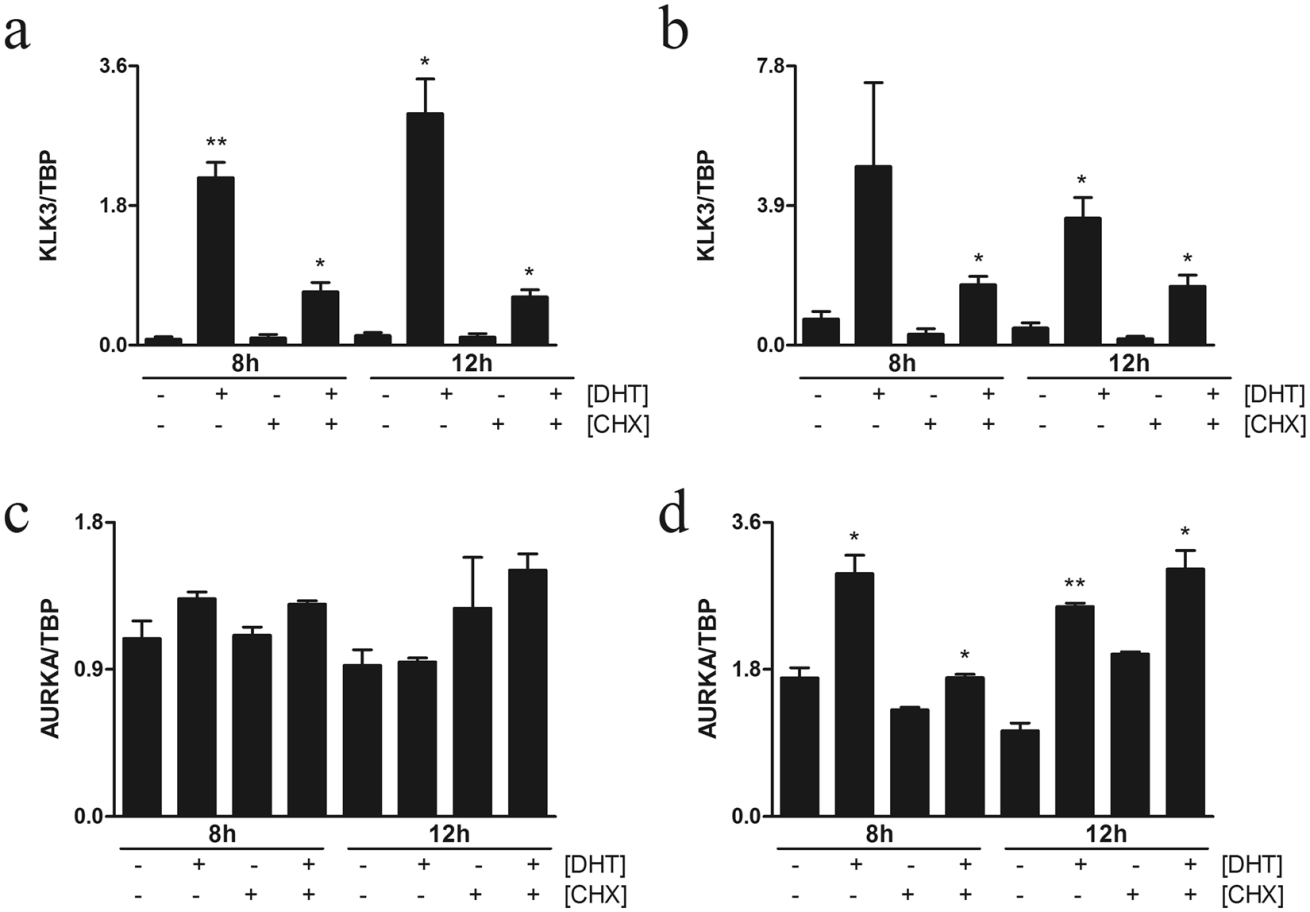
AR overexpression sensitizes *AURKA* under androgen regulation. LNCaP-cells were stably transfected to overexpress 8- to 10-fold higher wt-AR (LNCaP-ARhi) compared to that in empty vector LNCaP-pcDNA3.1 cells. Bar plots show the relative expression quantified with qRT-PCR and normalized to *TBP*. Relative *KLK3* expression in (**a**) LNCaP-pcDNA3.1 and (**b**) LNCaP-ARhi cells and relative *AURKA* expression in (**c**) LNCaP-pcDNA3.1 and (**d**) LNCaP-ARhi after 8 and 12 h of treatment with DHT (10 ng) with and without CHX (10 µg). *P < 0.05 and **P < 0.01, unpaired t test.

It has been previously shown that *AURKA* is overexpressed, especially in AR-negative NEPCs^6,7,25^. Interestingly, however, we found that its expression might be regulated by androgens in AR-overexpressing prostate cancer cells^18^ (Supplementary Figure S2). Therefore, we initially investigated whether *AURKA* is directly regulated by androgen in high AR-expressing (LNCaP-ARhi, described in ref.^18^) cells under dihydroxytestosterone (DHT) stimulation using cycloheximide (CHX) to stop translation, thereby inhibiting all downstream transcription events after stimulation. We grew both LNCaP-ARhi cells and the empty vector transfected control LNCaP (LNCaP-pcDNA3.1) cells with and without 10 nM DHT in the presence and absence of 10 µM CHX for 8 and 12 h. As expected, the expression of a well-known AR target gene *KLK3* (alias PSA) similarly increased in the presence and absence of CHX in both LNCaP-ARhi and empty vector transfected LNCaP-pcDNA3.1 cells (Fig. 1a,b). Whereas *AURKA* expression was significantly increased in the same conditions in LNCaP-ARhi cells, while it was not increased in LNCaP-pcDNA3.1 cells in the presence and absence of CHX (Fig. 1c,d). Since *AURKA* expression was increased despite the presence of CHX in LNCaP-ARhi alone, the data clearly suggest that overexpression of AR sensitizes *AURKA* to become directly androgen-regulated.

**Figure 2:**
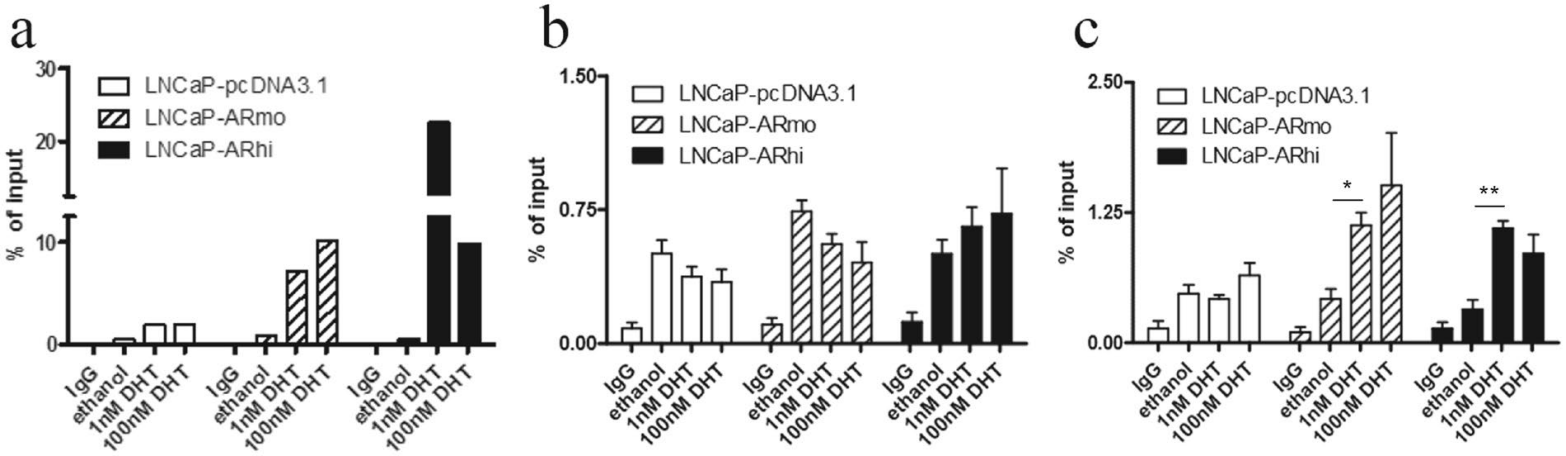
Androgen receptor (AR) binds to *AURKA*. (**a**) AR binding to the *KLK3*-enhancer (n = 1), (**b**) *AURKA*-promoter and (**c**) intronic region under DHT stimulation (n = 2). Traditional AR-ChIP-qPCR was performed after hormone starvation for 4 days followed by treatment with 1 and 100 nM DHT stimulation of LNCaP-pcDNA3.1 (control) and two independent AR-overexpressing cell lines (LNCaP-ARmo and LNCaP-ARhi). Bars show enrichment of AR binding expressed as the % of input. *P < 0.0232 and **P < 0.0107, unpaired t test.

To further validate the direct regulation of *AURKA* by androgens, we performed traditional ChIP-qPCR for ARBSs seen in the *AURKA* promoter and intronic region that have been recapitulated in several AR ChIP-seq datasets published by us and others^20–23^ (Supplementary Figure S3). First, we validated AR binding to the well-known enhancer region of *KLK3* in both a control LNCaP-pcDNA3.1 cell line and in two independent AR-overexpressing LNCaP-AR cell lines treated for 2 h with 1 nM and 100 nM DHT. We found significant enrichment of AR in all three cell lines with both DHT concentrations, as expected (Fig. 2a). Next, we measured AR binding on the *AURKA* promoter and intronic regions. The AR-binding on promoter region of *AURKA* was not significantly induced by androgens in any of the three cell lines (Fig. 2b). However, AR binding in the intronic region was significantly increased in AR-overexpressing cells under both 1 and 100 nM DHT stimulation compared to the empty vector transfected LNCaP-pcDNA3.1 cells (Fig. 2c). These results are concordant with the androgen-induced *AURKA* transcription in the presence of CHX in LNCaP-ARhi cells (Fig. 1d) and support the direct regulation of *AURKA* in cells that highly express AR.

**Figure 3:**
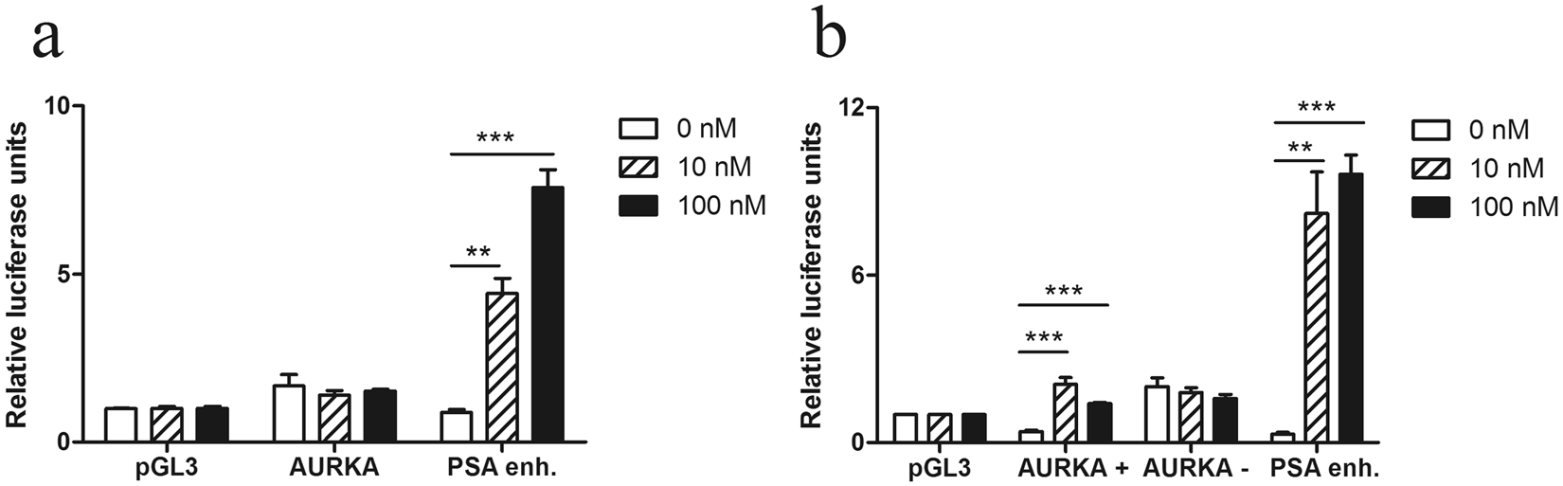
Activity of ARBS in promoter and intronic regions of *AURKA*. A luciferase assay was performed in LNCaP-ARhi cells with different DHT concentrations (0, 10 or 100 nM). Cells were transfected with pGL3-basic-LUC (LUC), pGL3-PSA5.8-LUC (PSA), pGL3-AURKApromoter-LUC (AURKA) and pGL3-AURKAenhancer-LUC (AURKA enh.), and luciferase activities were normalized to the renilla control plasmid (LUC). (**a**) Activity of AR binding to the *AURKA* promoter and positive (PSA) as well as negative control regions with different androgen stimulation. (**b**) Activity of AR binding to the *AURKA* enhancer and promoter with enhancer region and positive (PSA) and negative control (pGL3) regions with different androgen stimulation. *P < 0.05; n.s. = not significant. The means ± s.d. are shown.

To confirm the activity of the AR binding, we performed a transient luciferase reporter assay in LNCaP-ARhi cells under different DHT concentrations. We transfected the cells with *AURKA* promoter alone and the intronic regions in both the + and – orientations in the same construct. Empty luciferase vector and *KLK3* enhancer luciferase vector constructs were used as negative and positive controls, respectively (Fig. 3a). No androgen stimulated luciferase activity was found in the cells transfected with *AURKA* promoter construct alone (Fig. 3a), whereas the luciferase activity of the pGL3-*AURKA*promoter-enhancer(+)-LUC and PSA vectors were significantly increased when DHT was added (Fig. 3b). No change was found in the activity of pGL3-*AURKA*promoter-enhancer(−)-LUC vector. The data suggest that the intronic ARBS of *AURKA* is needed to induce androgen regulation in cells expressing high levels of AR.

**Figure 4:**
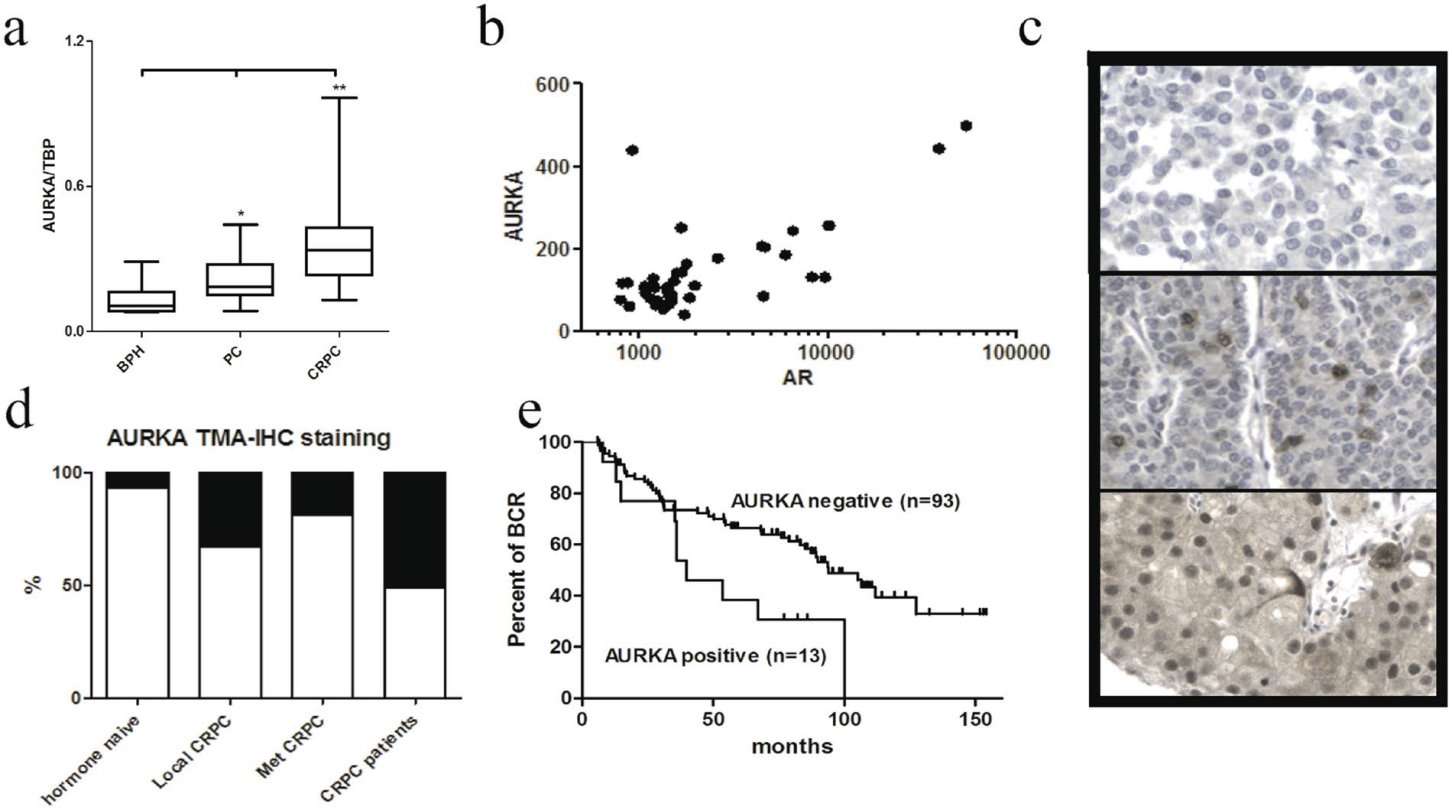
AURKA expression and correlation in clinical prostate cancer specimens. (**a**) AURKA expression (qRT-PCR) in an independent prostate cancer cohort of 8 BPH, 27 hormone-naïve prostatectomy prostate cancer patients and 16 CRPC specimens. *P = 0.038 and **P = 0.002, unpaired t test. (**b**) *AURKA* correlation with *AR* in 39 AR-positive prostate cancer specimens^19^. r(Pearson) = 0.751, p < 0.0001. (**c**) Representative AURKA IHC stains showing negative (upper panel) and positive stains with > 1% (middle) and ~80% (bottom) of prostate cancer cells. (**d**) Proportions of AURKA positive samples in hormone untreated prostate cancer, local CRPC, met-CRPC specimens and CRPC patients. (**e**) Kaplan-Meier analysis of biochemical recurrence (BCR) in hormone naïve prostate cancer patients. AURKA positive (with > 1% of the tumor cells, red line) patients had a significantly worse BCR rate. P-value = 0.017, Mantel-Cox test.

Next, we validated our previous RNA-seq data^19^ and studied *AURKA* expression in freshly frozen clinical prostate cancer cohort with traditional qRT-PCR. Previously, *AURKA* expression has been shown to increase more than four-fold, especially in the neuroendocrine type of PC (NEPC) fully negative for AR^6,26^, which was also observed in our data (Supplementary Figures S4 and S5). However, our study aim was to focus on the non-classical androgen regulation of *AURKA* in a high AR-positive cell context (Supplementary Figure S6). Therefore, all AR-negative NEPC type cancer specimens were excluded from our analysis (Supplementary Figures S4 and S5). The *AURKA* expression was significantly higher in both hormone naïve PC and CRPC (*P* = 0.038 and *P* = 0.002, respectively, Fig. 4a). We also observed a positive correlation between *AR* and *AURKA* expression in our AR-positive RNA-seq data (r = 0.751, p < 0.0001, Fig. 4b) as well as in the Taylor *et al*.^24^ microarray data set (r = 0.576, p < 0.0001, Supplementary Figure S7a) and 17 LuCaP-xenografts (r = 0.523, *P* = 0.03, Supplementary Figure S7b) after excluding AR-negative NEPC cases. In a bigger cohort of prostate cancer specimens^24^, the upper quartile of *AURKA* expression in 127 hormone-naïve, prostatectomy-treated cases was also significantly associated with biochemical recurrence (*P* = 3.09e-5, Supplementary Figure S7c).

Subsequently, we studied AURKA protein expression in clinical samples with immunohistochemistry (IHC) in FFPE tissue samples representing 106 hormone naïve prostatectomies, 126 locally recurrent CRPC samples and 104 CRPC metastases from 31 patients who died of prostate cancer. The staining was classified as positive when the staining intensity was 1 or higher in more than 1% of the cells in the particular specimen and negative when the staining intensity was zero in all of the cells or if only less than 1% of the cells were positive (Fig. 4c). All positive samples showed heterogeneous staining for AURKA, and only 5% of all samples had more than 50% AURKA positive cells. In local recurrent CRPC specimens and CRPC metastases, the frequency of AURKA positivity was significantly higher compared to hormone naïve prostate cancer samples (*P* < 0.0001 and *P* < 0.0001, respectively, Table 1, Fig. 4d). Positive AURKA protein expression was found in 12% (13/106) of hormone-naïve prostatectomy cancers, 47% (59/126) of local CRPC samples and 22% (23/104) of CRPC metastases (Table 1, Fig. 4d). Because the number of distinct metastases varied significantly patient-wise, we also analyzed how many patients had at least one AURKA positive metastasis. In our cohort, 23/31 patients had at least three metastases. Although the number of AURKA positive metastases was lower than the locally recurrent CRPC samples (22% and 47%, respectively), the number of patients who had at least one AURKA positive metastasis was similar, as seen in locally recurrent CRPC specimens of single patients. Therefore, of all CRPC patients with three or more metastatic specimens (12/23), 52% had at least one AURKA positive metastasis. All patients with three or more separate metastases in different organs had AURKA negative metastases.

**Table 1:** Association of clinicopathological variables. AURKA positivity was associated with clinico-pathological variables and staing of AR, Ki67, EZH2 and PTEN expression levels in AR positive pros-tate cancer samples (NEPCs were excluded from the analysis)

Variable	AURKA expression		*P*
	Negative	Positive	
Prostatectomy specimens, n (%)	93 (88)	13 (12)	
Locally recurrent CRPCs, n (%)	67 (53)	59 (47)	
Metastasized CRPCs, n (%)a	81 (78)	23 (22)	
Metastasized CRPC patients, n (%)^a^	11 (48)	12 (52)	***<0.0001***
Prostatectomy specimens:			
Gleason score, n (%)^a^			
<7	37 (92)	3 (8)	
7	42 (87)	6 (13)	
>7	8 (73)	3 (27)	*0.204*
pT Stage, n (%)^a^			
pT2	59 (88)	8 (12)	
pT3	31 (86)	5 (14)	*0.777*
AR expression, n (%)^b^			
weak or moderate	13 (87)	2 (13)	
strong	54 (83)	11 (17)	*0.504*
ERG expression, n (%)^b^			
negative or weak	48 (60)	8 (62)	
moderate or strong	32 (40)	5 (38)	*0.734*
PTEN expression, n (%)^b^			
negative or weak	40 (56)	7 (70)	
moderate or strong	31 (44)	3 (30)	*0.508*
p53 Copy number, n (%)^b^			
normal	48 (84)	4	
Heterozygous deletion	9	1	*1.000*
SPINK expression, n (%)^b^			
negative or weak	64 (84)	11 (100)	
moderate or strong	12 (16)	0 (0)	*0.349*
PSA ng/mL (mean +/− SD)^c^	14.3 (10.1)	15.4 (12.4)	*0.948*
Age (mean +/− SD)^c^	63.3 (4.8)	60.7 (5.9)	*0.126*
Ki-67 (mean+/− SD)^c^	5.4 (3.2)	7.7 (4.7)	***0.030***
EZH2 (mean +/− SD)^c^	22.9 (15.2)	23.2 (16.9)	*0.934*

^a^χ2 test.

^b^Fisher’s exact test.

^c^Unpaired t test.

In the hormone-naïve prostatectomy-treated patients, AURKA expression was found to be significantly associated (*P* = 0.017, Mantel-Cox test) with poor progression free-survival (Fig. 4e). The AURKA protein expression levels were not associated with any common clinicopathological variables, such as the age at diagnosis, primary PSA levels, pT-stage or Gleason score (*P* = 0.126, *P* = 0.948, *P* = 0.777 and *P* = 0.204, respectively; χ2-test and unpaired t-test; Table 1). AURKA positivity was significantly associated with MKI67 (Ki-67) staining (*P* = 0.030), but it was not associated with ERG, EZH2, PTEN or SPINK expression or with the TP53-copy number (*P* = 0.7324, *P* = 0.934, *P* = 0.508, *P* = 0.349 and *P* = 1.000, respectively; Table 1). By contrast, CRPC metastases had higher variation in the AR expression level, and the correlation of AURKA positivity was significantly associated with AR expression levels (*P* = 0.005, χ2-test) as well as with ERG positivity (*P* = 0.004, χ2-test; Table 2). AURKA positivity was also associated with the Ki-67 and EZH2 levels (*P* = 0.025 and 0.035, respectively; Mann-Whitney U test; Table 2). Similar to hormone naïve prostate cancer specimens, no association was found with PTEN, TP53 or SPINK expression (*P* = 0.420, *P* = 0.096 and *P* = 0.474, respectively; Fisher’s exact test; Table 2). In locally recurrent CRPC samples, AURKA protein expression was significantly associated with EZH2 expression (*P* = 0.032, Mann-Whitney U test, Table 2).

**Table 2:** Association of AR, EZH2, Ki67, PTEN and SPINK with AURKA in CRPC specimens. AURKA positivity was associated with staining of AR, Ki67, EZH2 and PTEN expression levels in AR positive CRPC samples (NEPCs were excluded from the analysis)

Variable	AURKA expression		*P*
	Negative	Positive	
Met-CRPCs:			
AR expression, n (%)^a^			
weak	31 (44)	4 (20)	
moderate	30 (43)	7 (35)	
strong	9 (13)	9 (45)	***0.005***
ERG expression, n (%)^b^			
negative	37 (60)	4 (20)	
positive	25 (40)	16 (80)	***0.004***
PTEN expression, n (%)^b^			
negative	42 (68)	11 (55)	
positive	20 (32)	9 (45)	*0.420*
p53 expression, n (%)^b^			
negative	40 (62)	16 (84)	
positive	25 (38)	3 (16)	*0.096*
SPINK expression, n (%)^b^			
negative	54 (87)	16 (80)	
positive	8 (13)	4 (20)	*0.474*
Ki-67 (mean +/− SD)^d^	9.3 (9.5)	14.5 (9.3)	***0.025***
EZH2 (mean +/− SD)^d^	27.9 (22.4)	39.6 (20.4)	***0.035***
Local CRPCs			
AR expression, n (%)^a^			
weak	1 (4)	1 (6)	
moderate	9 (39)	6 (38)	
strong	13 (57)	9 (56)	*0.073*
ERG expression, n (%)^b^			
negative	10 (38)	9 (56)	
positive	16 (62)	7 (44)	*0.344*
PTEN expression, n (%)^b^			
negative	6 (60)	7 (58)	
positive	4 (40)	5 (42)	*1.000*
SPINK expression, n (%)^b^			
negative	12 (86)	8 (67)	
positive	2 (14)	4 (33)	*0.365*
Ki-67 (mean +/− SD)^d^	14.4 (9.1)	18.1 (11.1)	*0.318*
EZH2 (mean +/− SD)^d^	39.2 (29.6)	58.9 (21.9)	***0.032***

^a^χ2 test.

^b^Fisher’s exact test.

^c^Unpaired t test.

**Figure 5:**
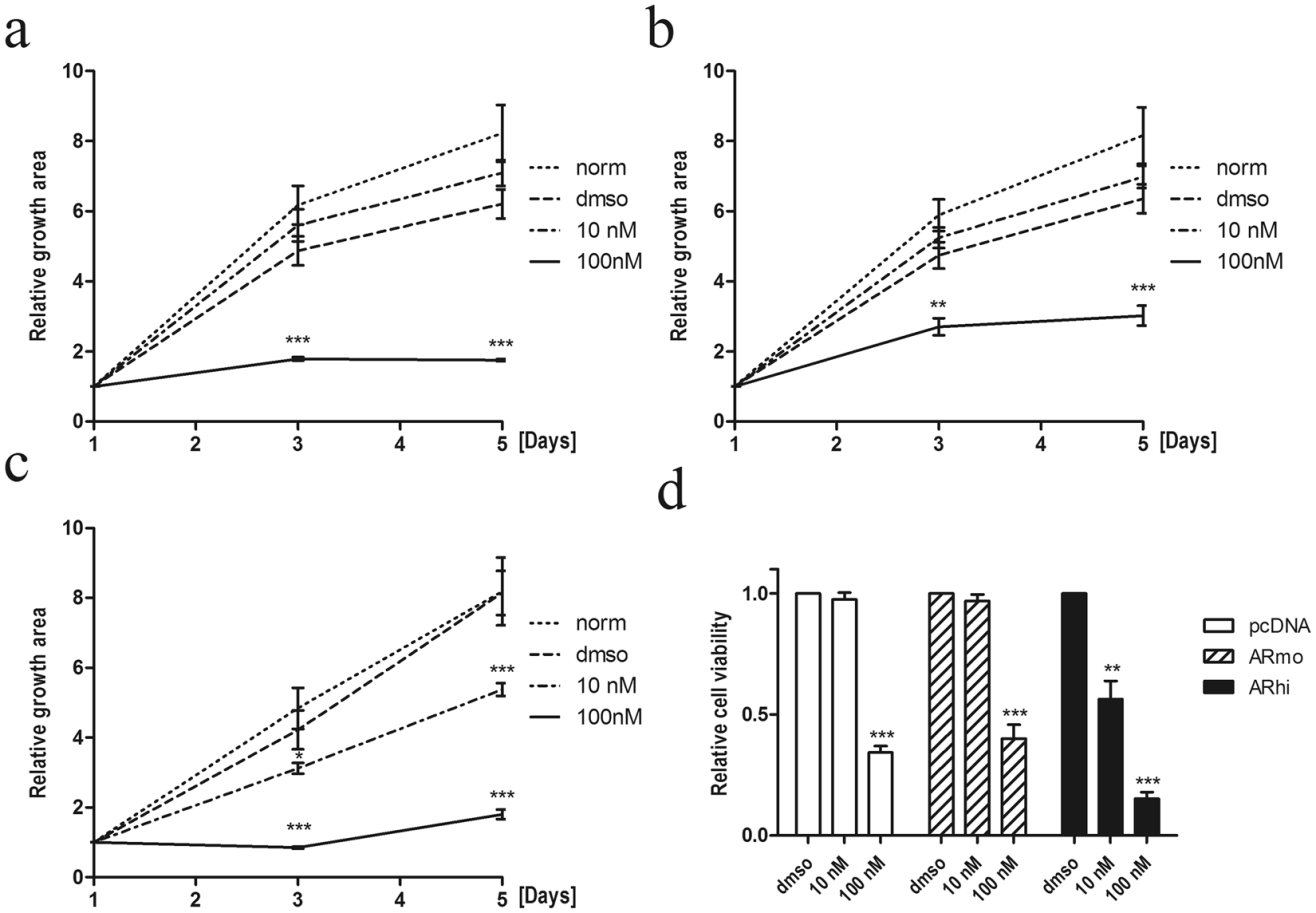
Relative growth of LNCaP-AR cells treated with the AURKA-specific inhibitor, alisertib. The relative growth of (**a**) LNCaP-pcDNA3.1, (**b**) LNCaP-ARmo, and (**c**) LNCaP-ARhi cells. All cells were equally seeded on 12-well plates and treated with vehicle (DMSO) or with 10 and 100 nM alisertib (MLN8237) in triplicate. The area of the attached cells in each well was measured daily and divided by the mean from day 1. (**d**) The relative cell viability of LNCaP-pcDNA3.1, LNCaP-ARmo, and LNCaP-ARhi cells at day 4 measured by Alamar blue and normalized to day 1 of each cell line. NT = no treatment. *p < 0.05, **p < 0.01, and ***p < 0.001, unpaired t test.

Finally, we wanted to test the effect of AURKA inhibition in high-AR expressing prostate cells (Fig. 5) under different concentrations of Aurora kinase inhibitor, alisertib (MLN8237), which is 200-fold more specific against AURKA than AURKB. First, to identify the optimal concentration to inhibit growth, we tested increasing concentrations (0–1000 nM) of alisertib and measured the growth of high AURKA expressing PC-3 cells^15^ (Supplementary Figure S8). The growth inhibition was 100% in PC-3 cells with a 100 nM or higher concentration of MLN8237. Thus, we treated LNCaP-pcDNA3.1 as well as LNCaP-ARmo and LNCaP-ARhi cells with 10 and 100 nM concentrations of alisertib to cause near maximal growth inhibition without overtreatment. All cell lines responded to 100 nM alisertib (Fig. 5) in a similar manner than AR-negative PC-3 cell line (Supplementary Figure S8). However, LNCaP-ARhi cells, expressing the highest levels of AR, were significantly more sensitive to AURKA inhibition because their growth was already reduced in the ten-fold lower concentration of alisertib compared to the LNCaP-pcDNA3.1 control cells (Fig. 5c and d). The modest 2- to 3-fold increase in the AR levels observed in LNCaP-ARmo cells was not sufficient to significantly sensitize cells to AURKA inhibition (Fig. 5c).

## Discussion

Our data indicate that *AURKA* is an androgen-regulated AR target gene. The regulation of *AURKA* specifically occurs in the context of highly AR expressing CRPC cells. We demonstrate that AR binds to the regulatory regions of the *AURKA* gene, and the *AURKA* transcript is upregulated in these cells. The increase in *AURKA* expression by androgens was also present despite the use of CHX that stops translation and inhibits possible secondary downstream effects of AR. We also validated the suggested prostate cancer specific AR binding by Pomerantz *et al*.^23^ into the intronic region of *AURKA* by ChIP-qPCR. The intronic AR binding was also responsive to enzalutamide treatment in the presence of DHT in highly AR expressing VCaP cells (^27^, Supplementary Figure S3), and our luciferase assay showed that the intronic element was responsive to the DHT stimulation. These results show that *AURKA* is an androgen-inducible gene in high AR expressing, androgen-sensitive prostate cancer cells.

We also report a transcriptional level correlation of *AURKA* and *AR* in clinical AR-positive prostate cancer specimens in two independent prostate cancer cohorts^19,24^. In CRPC metastases, AURKA positivity was significantly associated with high AR protein expression when AR-negative NEPC specimens were excluded from the analysis. AURKA expression was also associated with ERG positivity in these samples. The association with ERG could be explained in clinical samples by the binding of ERG to the *AURKA* promoter or enhancer region, promoting *AURKA* expression. It has been shown in VCaP cells that ERG binds to the *AURKA* promoter region 18 h after DHT treatment^28^. In the same study, no binding was observed 2 h after DHT treatment or without DHT stimulation^28^. Here, we used LNCaP-ARhi cells that are fully negative for ERG expression. Therefore, in our *in vitro* studies, the increased androgen stimulated *AURKA* expression cannot be explained by the ERG transcriptional regulation.

It has been shown that AURKA can function as an oncogene by enhancing the chromosome instability when it is overexpressed^16,29^. During mitosis and cytokinesis, AURKA associates with centrosomes and microtubules, affecting mitotic spindle formation and chromosome segregation^16,29^. AURKA overexpression is associated with centrosome amplification and aberrant mitotic divisions, leading to aneuploidy^29^. In our study, the frequency of AURKA positive staining was significantly increased in locally recurrent CRPC as well as metastatic CRPC cells compared to prostatectomy specimens, which is concordant with earlier studies that were performed by others^6,25,30^. The frequency of all metastasized CRPC samples (n = 104) from 31 individual patients was lower, which was similar to local CRPC specimens. However, our local CRPC specimens represent individual (single) patients and are thus not directly comparable with metastasis specimens. At a similar rate as that seen in local CRPC specimens, AURKA-positive metastases were found in 52% of the men who died of prostate cancer. Heterogeneity in AURKA expression in CRPC metastases from the same patients has not previously been reported. Different AURKA levels seen in different prostate cancer metastases may indicate variability in androgen sensitivity in the different metastases.

Previously, AURKA overexpression was reported in high-grade PINs, suggesting that it may be an early event in the development of prostate cancer^13,30^. We found that *AURKA* expression correlates with the *AR* level in hormone-naïve prostate cancer specimens, suggesting the *AURKA* expression may play a role in the early progression of prostate cancer. In agreement with the findings by Furukawa *et al*.^30^ and Beltran *et al*.^6^, we observed a significantly shorter biochemical recurrence rate in AURKA-positive prostate cancer specimens.

Here we also demonstrated that LNCaP-ARhi cells were significantly more sensitive to AURKA inhibition than cells expressing normal or modestly increased AR levels. Interestingly, Shu *et al*.^31^ demonstrated that AURKA can phosphorylate and activate AR, leading to a more active, potent form of AR. Recent results by Jones *et al*.^32^ also show that AURKA depletion reduces AR-V expression as well. Therefore, the data by us and others suggest that there may be a positive feedback loop between AR (both full-length and AR-Vs) and AURKA. On the other hand, using naïvely expressing AR cells, a study by Sarkar *et al*.^33^ found that AURKA is involved in proteosomal degradation of AR, thereby suggesting that inhibiting Aurora activity would promote prostate cancer growth by increasing AR levels and signaling. Thus, although more data should be gathered to support the use of AURKA inhibitors in primary tumours, where AR overexpression is not common, existing AURKA inhibitors could be useful in treating CRPC patients overexpressing full-length AR with or without AR-Vs, a finding that should be tested in future clinical trials.

Our findings on *AURKA* are in line with our previous data on the *CIP2A* oncogene^34^. We found that the expression of *CIP2A* is highly increased in AR-negative NEPC. Additionally, paradoxically, AR-overexpressing CRPCs express significantly more *CIP2A* than hormone-naïve PCs, although the expression levels are lower than those in NEPCs. In addition, the expression of *CIP2A* is increased with DHT stimulation of LNCaP-ARhi cells. Of note, the increased expression of *CIP2A* or *AURKA* upon androgen stimulation is not as high as with classical androgen stimulated genes, such as *TMPRSS2* and the *KLK*s (such as PSA). Taken together, these findings suggest that oncogenes that are upregulated in NEPC can also be upregulated, although to a lesser extent, in AR-positive CRPC. This implies that AR-positive CRPC and NEPC are not entirely different forms of the disease; instead, the AR-positive CRPC may acquire NEPC-like phenotypes.

In conclusion, we have demonstrated that *AURKA* is overexpressed not only in NEPC but also in the most common form of AR-positive CRPC. The expression of *AURKA* is androgen-regulated in such cells, and AURKA inhibition suppresses the growth of CRPC cells that express high levels of AR. Further studies are needed to determine whether CRPC patients would benefit from combined AURKA inhibition treatment and ADT.

## Materials and Methods

### Clinical tumor samples

The use of clinical material was approved by the ethical committee of the Tampere University Hospital (TAUH, Tampere, Finland) and the National Authority for Medicolegal Affairs and the Johns Hopkins Medicine Institutional Review Board (autopsy samples). Written informed consent was obtained from the subjects. All methods were performed in accordance with the relevant guidelines and regulations. Representative regions of formalin-fixed paraffin-embedded (FFPE) tissue blocks were chosen for tissue microarray and constructed as previously described^8^.

### Prostatectomy specimens

One hundred six FFPE prostate cancer samples from consecutive prostatectomies were obtained from TAUH. The clinicopathological variables of the cohort are given in Table 1. Progression was defined as a prostate-specific antigen (PSA) value of 0.5 ng/mL or more in two consecutive measurements or the emergence of metastases. Fifty-one percent of the patients experienced progression.

### Locally recurrent CRPC specimens and CRPC metastases

One-hundred and seventy-seven FFPE samples of locally recurrent castration-resistant prostate cancer (CRPC) from transurethral resection of the prostate (TURP) were obtained from the TAUH^8^. Ninety FFPE metastases were obtained from 31 men who died of CRPC and underwent autopsy as part of the project to Eliminate Lethal Prostate Cancer (PELICAN) rapid autopsy program at the Johns Hopkins Autopsy Study of Lethal Prostate Cancer^35^. During the course of treatment for metastatic prostate cancer, all subjects received androgen deprivation therapy either with an LHRH analogue or an orchiectomy. Many also intermittently received one or more antiandrogens during their disease course.

### Freshly frozen clinical prostate specimens

Fresh-frozen tissue specimens from 12 benign prostate hyperplasia (BPH) cases, 27 hormonally untreated prostate cancers and 14 local CRPCs were acquired from Tampere University Hospital (Tampere, Finland). Untreated prostate cancer samples were obtained by radical prostatectomy and locally recurrent CRPC samples by TURP. Samples were snap-frozen and stored in liquid nitrogen. All samples contained a minimum of 70% cancerous or hyperplastic cells.

### Prostate cancer cell lines and xenografts

PC-3 and LNCaP prostate cancer cell lines were obtained from the American Type Cell Collection (Manassas, VA, USA). LNCaP-ARhi, LNCaP-ARmo and LNCaP-pcDNA3.1 cell lines have been previously established in our laboratory^18^. Seventeen previously established LuCaP-series xenografts (LuCaP 23.1, 23.8, 23.12, 35, 41, 58, 69, 70, 73, 77, 78, 81, 86.2, 92.1, 96, 105 and 115)^36^ were provided by Prof. Robert L. Vessella (University of Washington, Seattle, WA, USA).

### Chromatin immunoprecipitation

Chromatin immunoprecipitation (ChIP) was performed as previously described^37^. Briefly, cells were hormone starved for 4 days and subsequently treated with DHT at different concentrations for 2 h. Cells were then fixed, pelleted and lysed. The chromatin was immunoprecipitated with 10 µg of normal rabbit IgG (Santa Cruz Inc., Santa Cruz, CA, USA) or 10 µl of AR antibody^21,22^. Immunoprecipitated DNA was purified using a QIAgen mini kit (Qiagen, Dusseldorf, Germany) and amplified using the following primers: for the *AURKA* promoter, reverse: ACCAGCTACTCTCCCCGTGT and forward: CGGTAGATTGGGCAGGATT; for the *AURKA* enhancer, reverse: CCCTTTCCGTGCTGTATTTC and forward: TGGCAATACTCCATCCACTCT; and for KLK3 enhancer, reverse: CCAGAGTAGGTCTGTTTTCAATCC and forward: TGGGACAACTTGCAAACCTG.

### Plasmid constructs and cloning

ARBS in the *AURKA* promoter region was cloned into the pGL3-Basic vector (Promega) containing the luciferase gene and multiple cloning region. PCR was performed for normal human leukocyte DNA to amplify the ARBS *AURKA* promoter. Amplified ARBS *AURKA* promoter was cloned between NheI and BglII sites. The primers used for PCR were as follows: forward primer ATGCAGAG**GC** **T** **A** **G** **C**TAAGGACT containing the NheI site, and reverse primer CGCCACTG**AGAT** **C** **T**CCCCCACG containing the BglII site (underlined letters indicate mutated components and bold letters indicate the restriction enzyme site).

ARBS in the AURKA enhancer region were cloned into the pGL3-Basic vector directly upstream of the *AURKA* promoter sequence. PCR was done for normal human leukocyte DNA to amplify the *AURKA* enhancer ARBS sequence. Amplified ARBS *AURKA* enhancer was cloned between KpnI and MluI sites in both orientations. The primers used for PCR were as follows: forward primer ( + orientation) GGGAGATCAGA**GGTA** **CC**GTGCATTCTTAT containing a KpnI site; reverse primer ( + orientation) AGTGGCCAAAGTTGCA**A** **C** **G** **CGT**TGAACCACAAAATAA containing a MluI site; forward primer (- orientation) CCCAGTGGCCAA**G** **GT** **AC** **C**AATGATCTGAACCA containing a KpnI site; and reverse primer (- orientation) ATTCTTATCACAT**AC** **GC** **G** **T**GATGTAAACTCTTA containing a MluI site (underlined letters indicate mutated components and bold letters indicate the restriction enzyme site).

### Luciferase assay

LNCaP-ARhi cells were grown in RPMI-1640 phenol-free medium (Biowhittaker, Lonza) with 10% charcoal/dextran-treated (CCS) FBS (HyClone, Thermo Scientific) and 1% Glutamine (Invitrogen Inc.) for four days before transfections and DHT stimulation. Cells were plated on a 48-well plate (100000–120000 cells/well). On the next day, they were transiently transfected with pGL3-basic (#E1751, Promega, Madison, USA), pGL3-PSA5.8-LUC (received from professor Jorma Palvimo, University of Eastern Finland, Finland^37^), pGL3-*AURKA*promoter-LUC, pGL3-*AURKA*promoter-*AURKA*enhancer(+)-LUC or pGL3-*AURKA*promoter-*AURKA*enhancer(-)-LUC. Each transfection was performed in four replicates. For normalization, control plasmid expressing the Renilla luciferase sequence was co-transfected into the cells. For Cos-7 cells, AR was co-transfected with the abovementioned vectors. Transfections were done with either Lipofectamine 2000 (Life Technologies) or jetPEI (Polyplus Transfection) transfection reagent according to the manufacturer’s instructions. DHT stimulation (0, 1, 10 or 100 nM) was added after transfection. After 24 h of transfection, the luciferase activity was measured with the Dual-Glo Luciferase assay system (Promega) according to the manufacturer’s instructions. Luminescence was measured with Envision Multilabel Reader (PerkinElmer). Measured luminescence levels were normalized to the renilla luminescence levels.

### Growth analysis

PC-3, LNCaP-pcDNA3.1 and LNCaP-ARhi cell lines were first equally seeded (15,000 cells/well) on 24-well plate as four replicates and were allowed to adhere overnight. On day 1, the cells were treated with DMSO (vehicle) or with appropriate concentrations (1–1000 nM) of MLN8237 (Selleck Chemicals LLC) in DMSO. All wells were scanned daily using the Surveyor Software (Objective Imaging Ltd.) with a camera (Imaging Inc., Canada) attached to the Olympus IX71 (Olympus, Tokyo, Japan) microscope, and the area of the attached cells in each well was counted by analysis with ImageJ Software (Wayne Rasband, National Institutes of Health, Bethesda, MD). Finally, the measured growth area of each well was divided by the mean area of day 1 for each following day.

### Immunohistochemistry

Antibodies against AURKA (1:50, NCL-L-AK2, Novacastra), ERG (EPR3864, Epitomics, Inc.), Ki-67 (MM1, Leica Biosystems Newcastle Ltd.), EZH2 (NCL-L-EZH2, Novacastra) and AR (1:200, 318, Novocastra Laboratories Ltd.) were used with a Power Vision + Poly-HRP IHC kit (ImmunoVision Technologies Co.) according to the manufacturer’s instructions. The protocol has been previously described by Leinonen *et al*.^38^ and for AURKA by Staff *et al*.^17^. Aperio ScanScope XT scanner (Aperio Technologies, Inc.) was used to scan the slides. Scoring was done in a blinded fashion with the use of virtual microscope^39^. Ki-67 and EZH2 scoring was performed with a web based application, Immunoratio^40,41^. AR staining was evaluated from 0 to 3, AURKA and ERG staining was evaluated as positive or negative.

### Statistical analyses

The Mann-Whitney U, χ2, Fisher’s exact and unpaired t tests were used to analyze the association between AURKA protein expression and clinicopathological variables, AR, ERG, Ki-67 and EZH2. Kaplan-Meier survival analysis and the Mantel-Cox test were used to determine the progression-free survival of patients. The unpaired t-test was used to calculate the difference in proliferation for growth curve analyses. The Mann-Whitney U test was used to determine the significant difference in luciferase activity.

### Data availability statement

All data generated or analyzed during this study are included in this published article (and its Supplementary Information files).

## Electronic supplementary material

Supplementary Information
