# Supplementary material for: The expression of AURKA is androgen regulated in castration-resistant prostate cancer
Source: Sci Rep. 2017 Dec 21;7:17978. doi: 10.1038/s41598-017-18210-3 (PMC5740165; doi:10.1038/s41598-017-18210-3)
Supplement: Supplementary file 1 — Supplementary Information [file 41598_2017_18210_MOESM1_ESM.pdf]

Supplementary information

The expression of *AURKA* is androgen regulated in castration-resistant prostate cancer.

Kati Kivinummi*, Alfonso Urbanucci*, Katri Leinonen, Teuvo L.J. Tammela, Matti Annala, William B. Isaacs, G. Steven Bova, Matti Nykter and Tapio Visakorpi

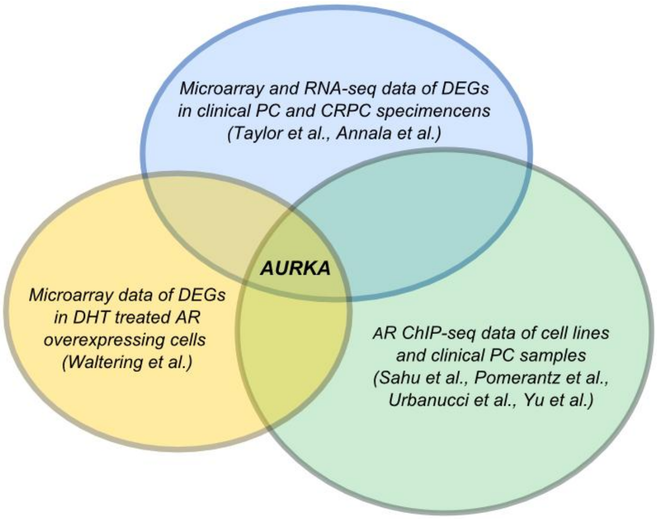

Supplement Figure 1. Illustration of the data sets that were used prior to the study. *AURKA* was found to be overexpressed (>2-fold) in clinical CRPC specimens compared to the hormonally untreated prostatectomies (Taylor *et al.*2010: n(PC)=125, n(CRPC)=18; Annala *et al*. 2015: n(PC)=27, n(CRPC=12), increased (> 2-fold) in AR-overexpressing cells LNCaP-ARhi and VCaP cells and DHT stimulated in LNCaP-ARhi cells (Waltering *et al.* 2009) and carried an ARBSs in ChIP-seq data sets of VCaP, LNCaP and LNCaP-ARhi cells (Yu *et al*. 2010, Sahu *et al.* 2011, Urbanucci *et al.* 2012) that were cancer specific in clinical prostate tissue specimens (Pomerantz *et al.* 2015). DEG = differently expressed genes, ChIP-seq = chromatin immunoprecipitation sequencing.

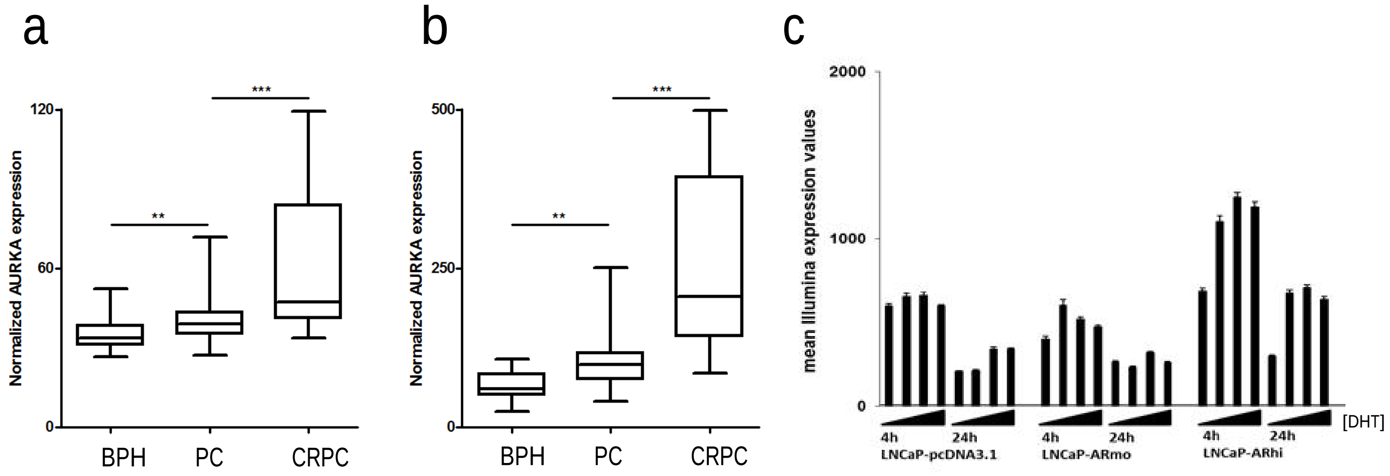

Supplementary Figure 2. *AURKA* expression in prostate cancer and CRPC specimens and after androgen stimulation in high-AR overexpressing cells. A) *AURKA* expression in a microarray (Affymetrix Human Exon 1.0 ST) data set of clinical samples of BPH (n=28), hormone naïve PC (n=125) and metastasis CRPC (n=18) specimens (Taylor *et al*. 2010). B) *AURKA* expression in an RNA-seq (Illumina HiSeq™ 2000) data set of clinical samples of BPH (n=12), hormone naïve PC (n=27) and local CRPC (n=12) specimens (Ylipää *et al*. 2015). AR-negative, neuroendocrine PC (NEPC) samples were excluded from both cohorts. C) *AURKA* expression after 0, 1, 10 and 100 nM DHT stimulation for 4 and 24 h to cells carrying different levels of AR. LNCaP-pcDNA3.1, LNCaP-ARmo 3-5x > AR, and LNCaP-ARhi 8-10x > AR-level compared to LNCaP-pcDNA3.1

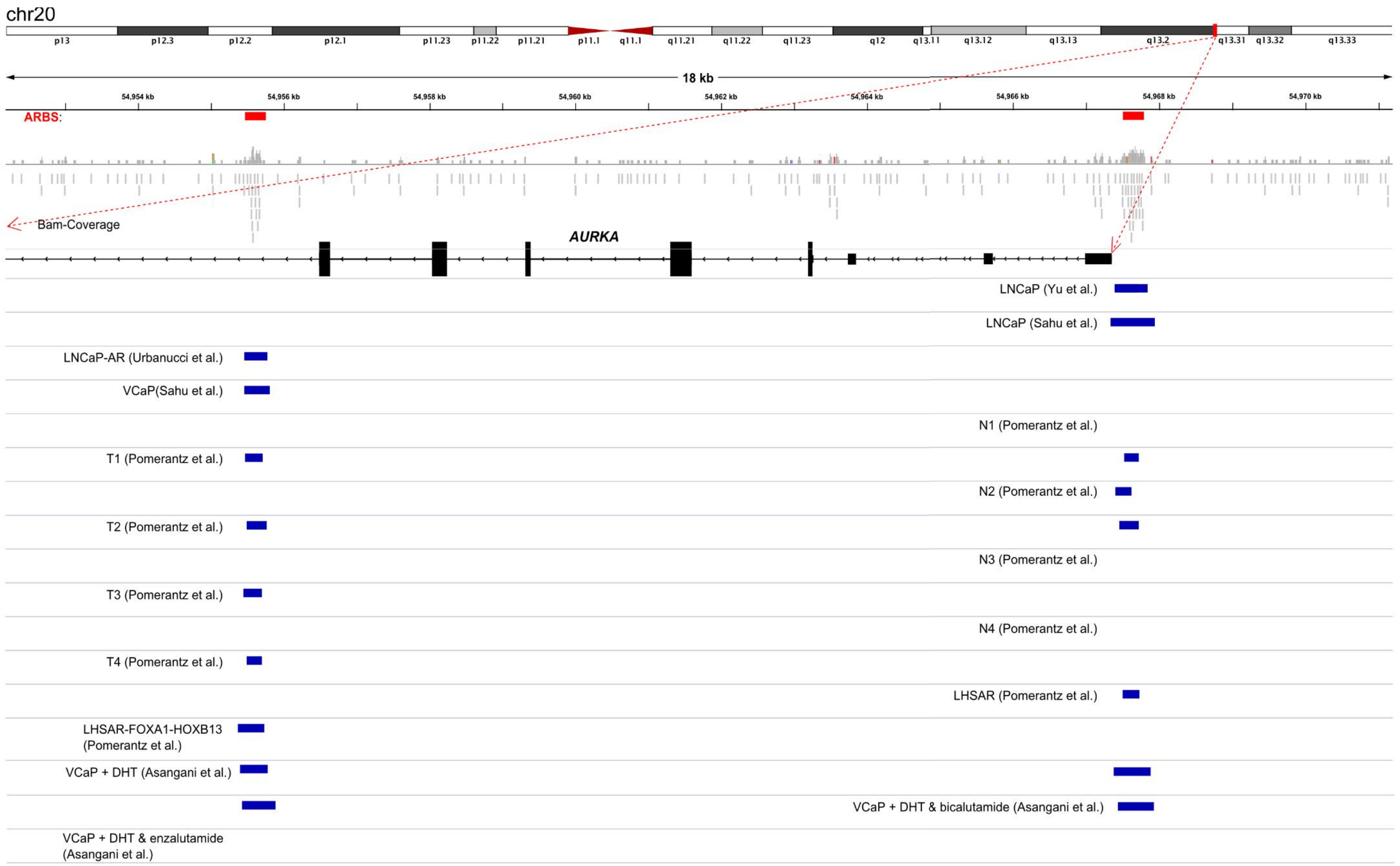

Supplementary Figure 3. AR binding on *AURKA*. Partial exonic structure of *AURKA* (black boxes represent the 5'UTR and exons) with AR binding sites (ARBS) from published AR ChIP-seq data from cell lines and clinical specimens. Two ARBSs found in different data are summarized with red boxes above the genetic structure of *AURKA*, and the ARBSs found in individual samples are illustrated with blue boxes above the structure. Each line represents an individual AR ChIP-seq sample. LNCaP and LHS-AR cells harbored ARBS in the promoter of *AURKA* (Yu. et al 2010, Sahu *et al*. 2011, Pomerantz *et al*. 2015) are shown in the first two and 3rd last rows under the schematic illustration of *AURKA*. High AR expressing VCaP and LNCaP-ARhi cells contained ARBS in the intronic region of *AURKA* (3rd and 4th row, Sahu *et al*. 2011, Urbanucci *et al*. 2012). ARBSs by Pomerantz *et al.* (2015) from paired uncancerous (N) and cancerous clinical specimens (T) are shown in rows 5-12. T1-4 represent tumor tissues and N1-4 represent normal adjacent tissues of the corresponding prostate cancer specimen. ARBS found in DHT, bicalutamide and enzalutamide treated VCaP cells are shown in rows 13-15 (Asangati et al. 2017).

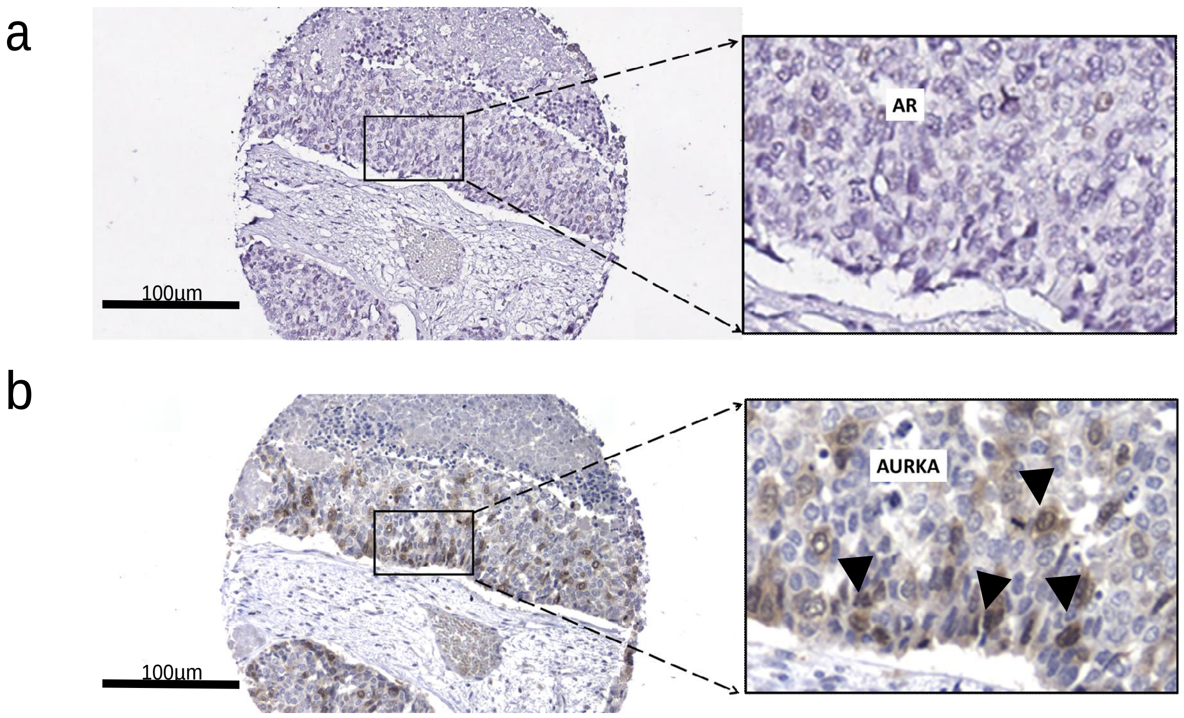

Supplementary Figure 4. AR and AURKA expression in metastasis NEPC specimen. IHC staining for A) AR (1:200, 318 monoclonal antibody, Novacastra) and B) AURKA expression (1:50, NCL-L-AK2 monoclonal antibody, Novacastra). AURKA-positive cells are highlighted with arrows in the zoomed image of a 20X objective magnification of the original TMA spot of the same NEPC specimen.

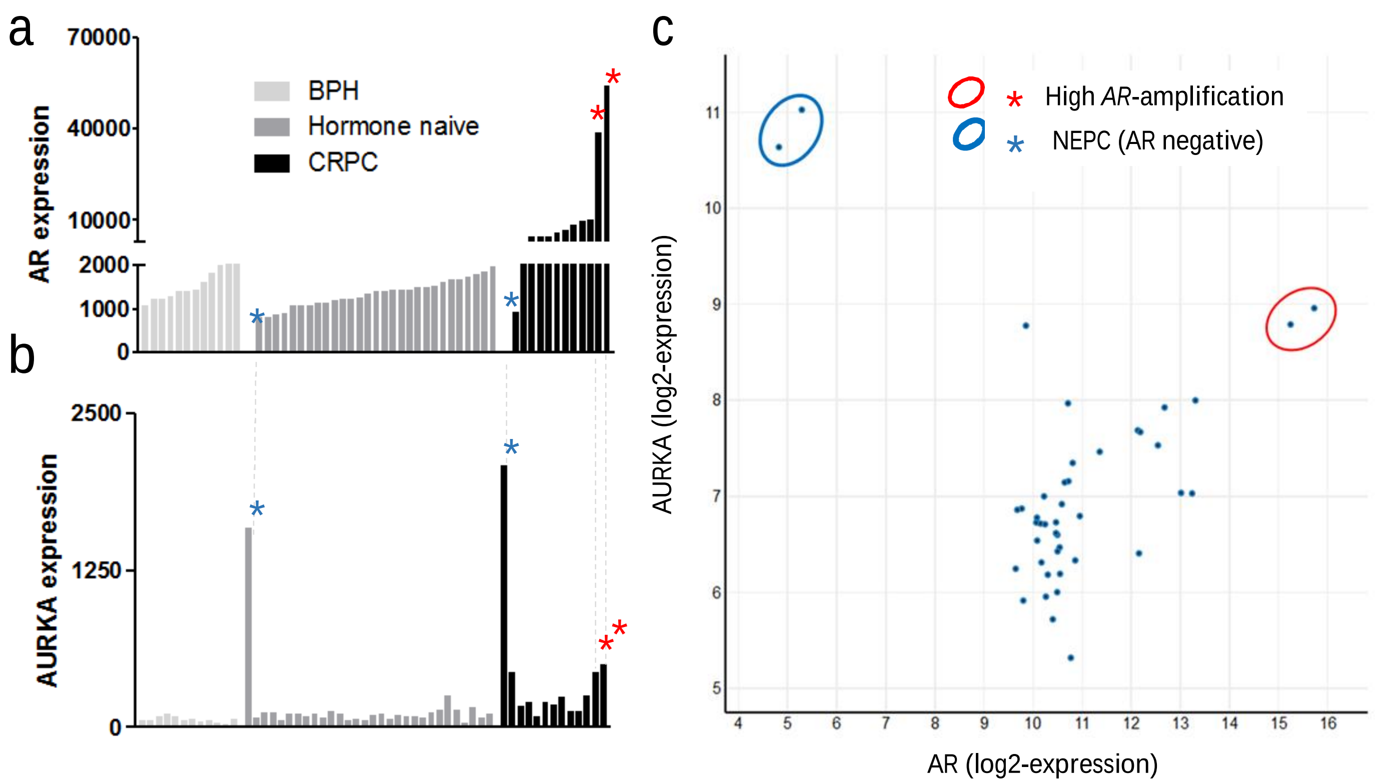

Supplementary Figure 5. *AR* and *AURKA* expression in clinical prostate cancer specimens. 12 BPH (light gray), 28 hormone naïve prostate cancer (dark gray) and 13 local CRPC (black) specimens were sequenced using an Illumina HiSeq 2000 platform (Ylipää *et al.* 2015). A) Normalized *AR* expression values in increasing order and B) normalized *AURKA* expression values in the same sample order for all three sample groups. Red asterisks indicate samples with a high level of amplification and expression of *AR* and blue asterisks indicate AR-negative NEPC samples. C) Two-gene scatterplot for AURKA and AR expression as 2-log expression values from the same clinical specimens as in A and B. The x and y-axes represent normalized 2-log expression values. Two NEPC samples are highlighted with a blue circle, and the high AR amplification carrying CRPC samples is highlighted with a red circle.

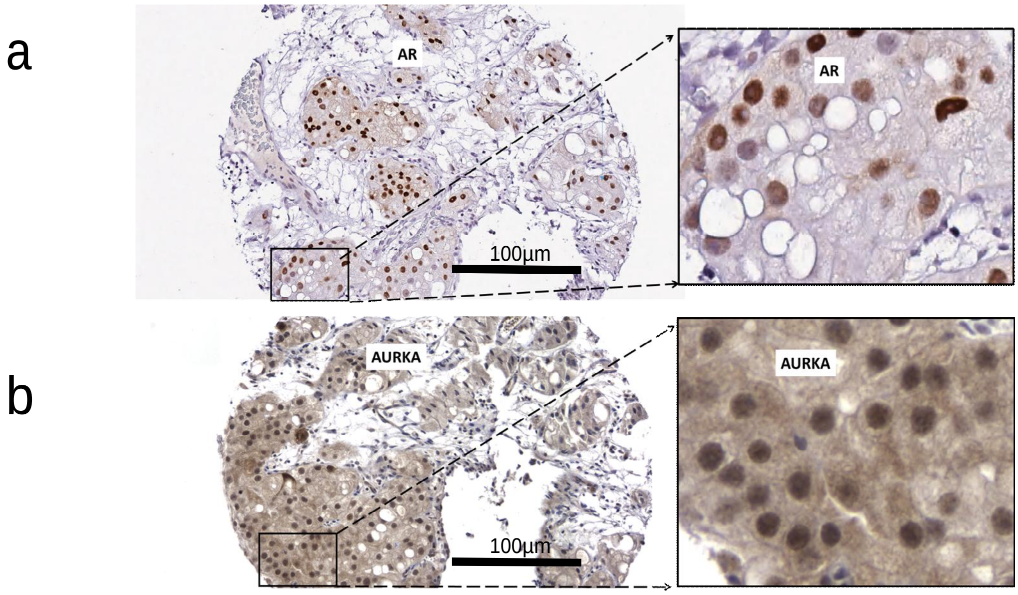

Supplementary Figure 6. AURKA expression in the CRPC sample with high level *AR* amplification. Example of IHC staining for AR amplification and overexpression carrying CRPC TURP specimen. A) AR (1:200, 318 monoclonal antibody, Novacastra). B) AURKA staining (1:50, NCL-L-AK2 monoclonal antibody, Novacastra).

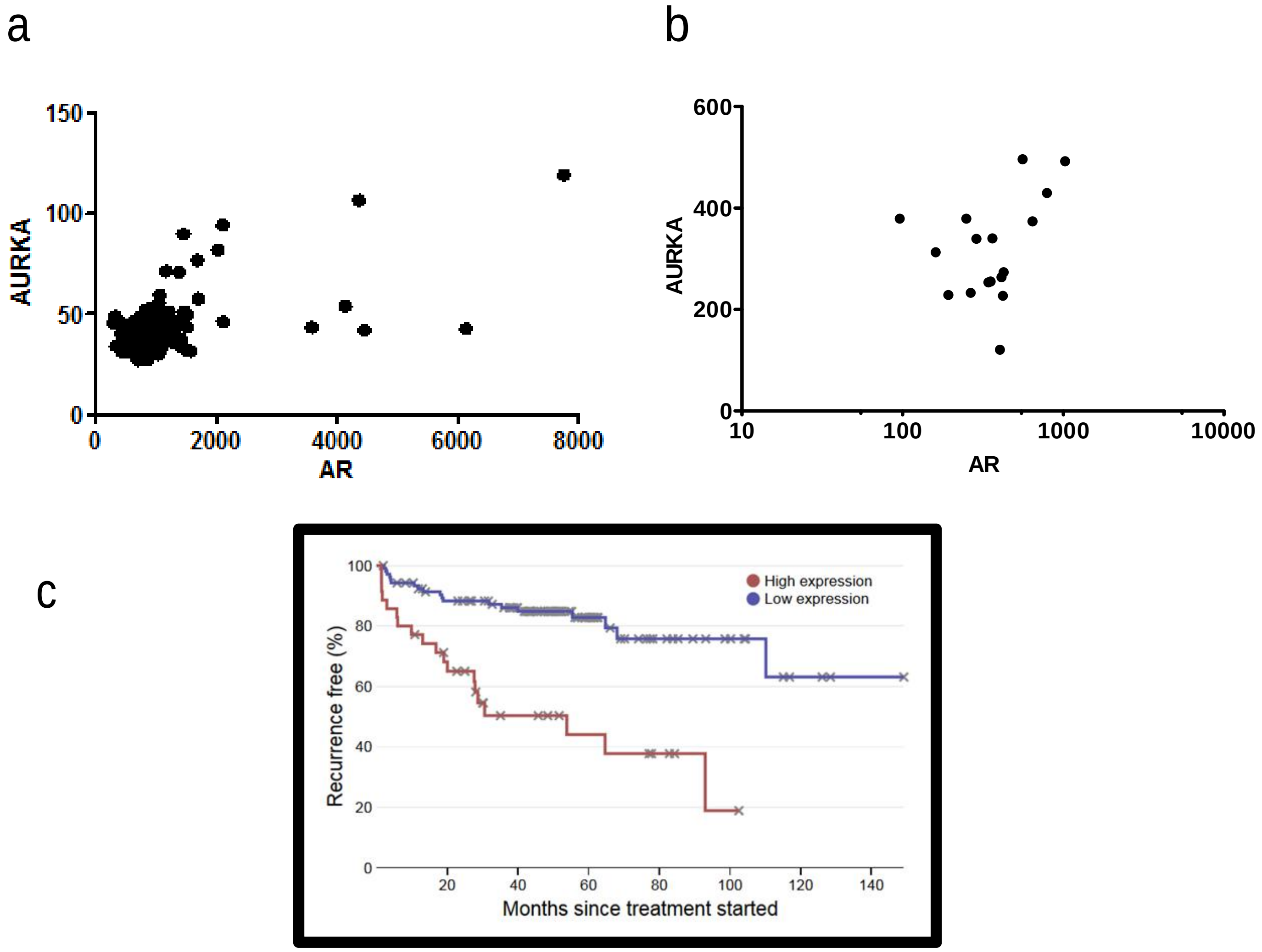

Supplementary Figure 7. *AURKA* correlation and association with progression in prostate cancer specimens. *AURKA* expression correlates with *AR* expression in microarray data of A) 143 clinical prostate cancer by Taylor *et al.* (2010) and in B) 17 LuCaP xenograft specimens after excluding AR-negative NEPC cases from the data. C) *AURKA* expression is associated with biochemical recurrence. Kapplan-Meier plot of 127 hormonally untreated prostate cancer specimens by Taylor *et al*. 2010 (Affymetrix Human Exon 1.0 ST array data) showing a significant association (P < 0.0001, Mantel-Cox test) for the upper quartile AURKA expression with the biochemical recurrence of prostate cancer.

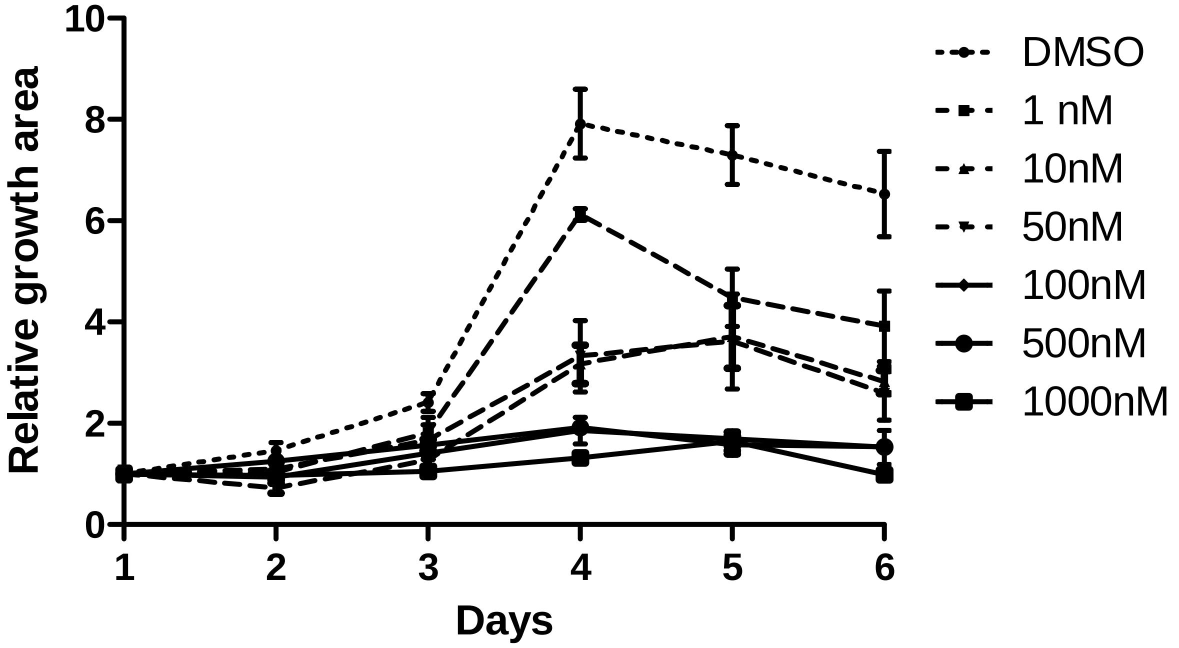

Supplementary Figure 8. Relative growth of PC-3 cells treated with increasing concentration of MLN8237. PC-3 cells were seeded (15 000 cells/well) on 24-well plate as four replicates. On day 1, the cells were treated with DMSO or with appropriate concentrations (1-1000 nM) of AURKA inhibitor (MLN8237 Selleck Chemicals LLC) supplemented to the normal growth medium. The area of the attached cells in each well was measured daily and divided by the mean from day 1.

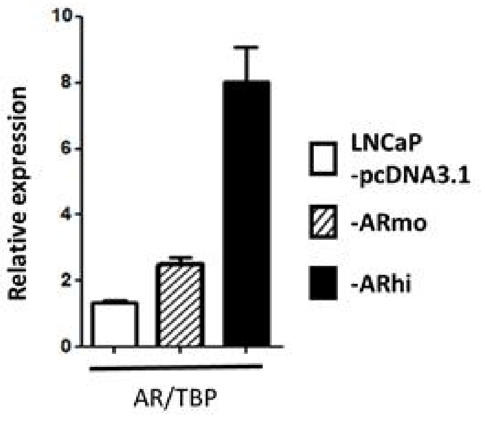

Supplementary Figure 9. Relative *AR* expression of the AR-overexpression cell line model. *AR* expression levels of LNCaP-pcDNA3.1 (white bars), -ARmo (striped bars) and -ARhi cells (black bars) quantified with qRT-PCR and normalized to *TBP*.

Supplementary Table S1. ARBS of upregulated novel AR target genes by Waltering *et al.* [1]

Gene name	Kaplan-Meier analysis (P value) [2]	ARBS by Yu et al. 2009 [3]	ARBS by Sahu et al. 2010 [4]	ARBS by Urbanucci et al. 2011 [5]	ARBS by Pomeranz et al. 2016 [6]	Cancer specificity by Pomeranz [6]
ASF1B	ns	-	-	-	-	
ATAD2	ns	Intron 1, +4.2 kb, Enh -9.0 kb	Intron 1, +4.2 kb, Enh -9.0 kb	Enh -9.0 kb	Intron 1 (+4.2 kb), Enh -9.0 kb	Yes , No
AURKA	3.09E-05	PP	PP, Intron 8 (+11.6 kb)	Intron 8 (+11.6 kb)	PP, Intron 8 (+11.6 kb)	No , Yes
AURKB	ns	Enh -9.6 kb	-	-	Enh -9.6 kb, Enh +36.9 kb	Yes, No
BUB1B	0.00114	PP	PP	-	-	
C19orf48	ns	Enh -37.7 kb	Enh -37.1 kb	Enh -37.7 kb	Enh -37.7 kb	No
CCNA2	ns	-	-	-	-	
CCNB2	ns	Enh +50 kb	Enh +50 kb	-	Enh +50 kb, Enh 29.8 kb	Yes, No
CDC20	ns	-		-	-	
CDC25A	ns	Enh -44 kb, -48.5 kb, -54.3 kb	Enh -44 kb, -48.5 kb, -54.3 kb	Enh -44 kb, -48.5 kb	Enh -44 kb, -48.5 kb, -54.3 kb	No, No, Yes
CDCA3	ns	PP (-0.3 kb)	PP (-0.3 kb)	PP (-0.3 kb)	-	
CDCA4	ns	Enh +41 kb	Enh +41 kb	-	-	
CDCA5	ns	Enh -11,9 kb, -20kb	Enh -11,9 kb, -20kb	-	Enh -20kb	Yes
CDCA7	ns	Enh +14.2 kb	Enh +14.2 kb	-	Enh +14.2 kb	Yes
CDK1	ns	Enh -10.2 kb, +39.0 kb	Enh -20.5 kb	-	Enh +39.0 kb	No
CDK2	ns	Enh -57.0 kb	Enh -57.0 kb	Enh -57.0 kb	Enh -57.0 kb, +46.9 kb	No , Yes
CDKN3	0.0145	Enh -63.9 kb	Enh -63.9 kb	-	-	
CENPM	ns	-	-	-	-	
CKS1B	ns	-	Enh + 42.4 kb	-	Enh + 42.4 kb	No
DLGAP5	0.0238	Enh -50 kb, +61 kb	Enh -50 kb, +61 kb	-	Enh -50 kb, +61 kb	No , No
DTL	ns	Enh -18.7 kb, -30.0 kb	Enh -30.0 kb	-	Enh -18.7 kb, -30.0 kb	Yes, Yes
FAM83D	ns	-	-	-	-	
FANCI	ns	-	-	-	-	
FEN1	ns	Enh -97 kb, +28.3 kb, +37.8 kb	Enh -97 kb, +28.3 kb, +37.8 kb	Enh -97 kb	Enh -97 kb, +16.0 kb, +37.8 kb	No
GINS2	ns	Intron 4, 7.4 kb	Intron 4, 7.4 kb	-	Intron 4, 7.4 kb	No
HMGB2	0.0457	Enh -37.6 kb	Enh -37.6 kb	-	-	
HMGCS2	0.0000862 *)	PP, Intron 1, +2.2 kb / Enh -9.0 kb	PP, Intron 1, +2.2 kb / Enh -9.0 kb	-	Intron 1, +2.2 kb / Enh -9.0 kb	Yes , Yes
HMMR	ns	Enh +22.8 kb	Enh +22.8 kb	-	Enh +22.8 kb	No
IQGAP3	ns	Enh -44 kb, Int +12.5 kb, +61.5 kb	Enh -44 kb, Int +12.5 kb, +61.5 kb	Enh -44 kb	Enh -44 kb, Int +12.5 kb, +61.5 kb	No, No, No
KIF20A	ns	Enh -24 kb, +20 kb	Enh -24 kb, +20 kb	Enh +20 kb	Enh +20 kb	No
MAD2L1	ns	Enh -19.7 kb	Enh -19.7 kb	-	-	
MCM4	ns	-	-	-	Enh -17.9, Intron 9 (+84 kb)	Yes, Yes
MELK	0.0175	-	-	-	-	

NCAPG	0.0015	-	-	-	-	
NDC80	ns	Enh -38 kb	Enh -38 kb	Enh -38 kb	Enh -38 kb	Yes
NMU	ns	-	-	-	-	
NUSAP1	ns	Intron 1 (+4.9 kb)	Intron 1 (+4.9 kb)	-	-	
PARPBP	0.0498	Enh -27.9 kb, -58.5, +80.0 kb	Enh -58.5, +80.0 kb	Enh +80.0 kb	Enh -27.9 kb, -58.5, +80.0 kb	No , No , Yes
PCNA	ns	Enh +26.3 kb	Enh +26.3 kb	Enh +26.3 kb	Enh +26.3 kb	Yes
PIMREG	ns	Enh +7.0 kb	Enh +7.0 kb	-	-	
PRC1	ns	Enh -47.8 kb	Enh -47.2 kb	-	Enh -47.8 kb	No
PRIM1	ns	Enh -6.9 kb	Enh -6.9 kb	Enh -6.9 kb	Enh -6.9 kb	No
PTTG1	ns	-	-	-	-	
RAD51AP1	ns	Enh +48 kb, -32 kb	Enh +48 kb, -32 kb	-	Enh -32 kb	Yes
RAD54L	ns	-	-	-	-	
RNASEH2A	ns	-	-	-	Enh -13 kb	Yes
SPAG5	ns	-	-	-	-	
STIL	ns	Intron (+46.6 kb)	Intron (+46.6 kb)	-	Enh -9.7 kb, -34.1 kb, Intron (+46.6 kb)	Yes, Yes, Yes
TK1	ns	intron 3, +2.0 kb Enh -11.8 kb, -24 kb	Enh -11.8 kb, -24 kb	Enh -27 kb		No
TOP2A	ns	-	-	-	-	
TYMS	ns	-	-	-	-	
UBE2C	0.00189	-	-	-	Enh -33.2 kb	No
UHRF1	ns	-	Enh +30 kb	-	Enh +30 kb	No
ZWINT	0.0129	Enh -22.6 kb, -39.7 kb	Enh -22.6 kb, -39.7 kb	Enh -22.6 kb	Enh -22.6 kb, -39.7 kb	No, Yes

ns = non significant (P>0-05)

*) = associated with decreased expression

ARBS = Androgen Receptor Binding Site

PP = Proximal Promoter

Enh = Enhancer

Kaplan-Meier analysis in the first column is analyzed from Taylor *et al*. [1] data of hormone naive prostate cancer specimens (n = 141).

“yes” and “no” in the last column indicate whether the ARBS, in order of appearance in the other columns, is prostate cancer specific according to Pomerantz *et al*. [5].

References:

[1] Waltering, K. K. et al. Increased expression of androgen receptor sensitizes prostate cancer cells to low levels of androgens. Cancer Res 69, 8141-8149 (2009).
[2] Taylor, B. S. et al. Integrative genomic profiling of human prostate cancer. Cancer Cell 18, 11-22 (2010).
[3] Yu, J. et al. An integrated network of androgen receptor, polycomb, and TMPRSS2-ERG gene fusions in prostate cancer progression. Cancer Cell 17, 443-454 (2010).
[4] Sahu, B. et al. Dual role of FoxA1 in androgen receptor binding to chromatin, androgen signalling and prostate cancer. EMBO J 30, 3962-3976 (2011).
[5] Urbanucci, A. et al. Overexpression of androgen receptor enhances the binding of the receptor to the chromatin inprostate cancer. Oncogene 31, 2153-2163 (2012).
[6] Pomerantz, M. M. et al. The androgen receptor cistrome is extensively reprogrammed in human prostate tumorigenesis. Nat Genet 47, 1346-1351 (2015).
[7] Asangani IA, Wilder-Romans K, Dommeti VL, Krishnamurthy PM, Apel IJ, Escara-Wilke J, Plymate SR, Navone NM, Wang S, Feng FY, Chinnaiyan AM. BET Bromodomain Inhibitors Enhance Efficacy and Disrupt Resistance to AR Antagonists in the Treatment of Prostate Cancer. *Mol Cancer Res* 14, 324-231 (2016).
